# Supplementary material for: Severity Patterns in COVID-19 Hospitalised Patients in Spain: I-MOVE-COVID-19 Study
Source: Viruses. 2024 Oct 30;16(11):1705. doi: 10.3390/v16111705 (PMC11598861; doi:10.3390/v16111705)
Supplement: Supplementary file 1 [file viruses-16-01705-s001.zip › viruses-3237292-supplementary.pdf]

viruses

Supplementary

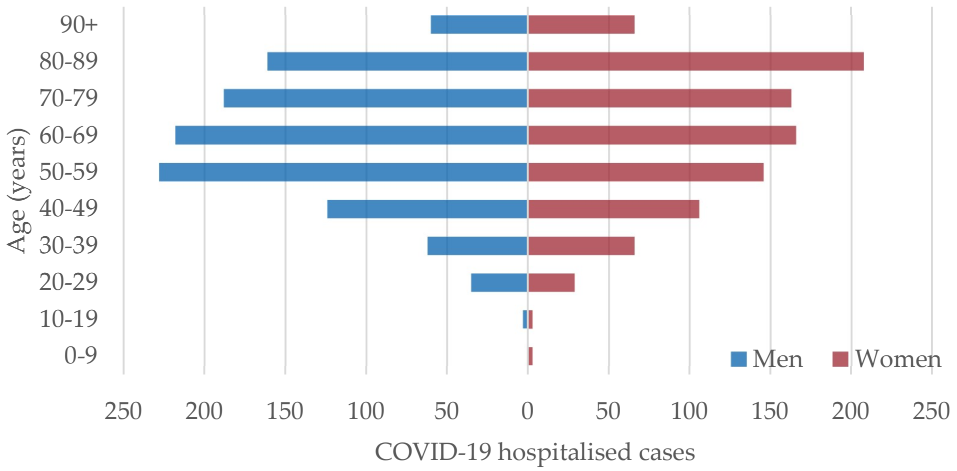

Figure S1. Spanish COVID-19 hospitalised cases by age and sex (I-MOVE-COVID19 study).

Table S1. Factors associated with death in Spanish COVID-19 hospitalised patients (I-MOVE-COVID19 study).

	**Dead** 309 (15.1%)	**Alive** 1740 (84.9%)	**p value**	**Crude OR (IC95%)**
**Sociodemographic and clinical factors**				
Age, grouped (0-39; 40-64; 65-84; 85+ years)			<0.001	0.00 (6.6e-4 – 0.03)
Age 0-39 years	1 (0.3)	214 (12.3)		(reference)
Age 40-64 years	38 (12.3)	772 (44.4)	0.020	10.53 (1.44-77.00)
Age 65-85 years	142 (46.1)	580 (38.3)	<0.001	52.4 (7.30-376.04)
Age 85+ years	127 (41.2)	174 (10)	<0.001	156.19 (21.66-1126.38)
Sex (men)	171 (55.3)	918 (52.8)	0.402	1.11 (0.87-1.42)
Centre (HUVN)	126 (40.8)	706 (40.6)	0.947	1.01 (0.79-1.29)
COVID-19 vaccinated	182 (20.3)	33 (26.0)	0.140	1.38 (0.90-2.12)
Flu vaccinated	611 (35.4)	182 (59.9)	<0.001	2.72 (2.12-3.49)
Number of previous hospitalisations (last year), mean ± SD	3.3 ± 4.7	2.2 ± 2.3	0.189	1.10 (0.95-1.30)
Number of previous medical visits (last year), mean ± SD	10.2 ± 7.9	6.3 ± 6.9	<0.001	1.06 (1.03-1.09)
Previous medical visits last year >12	25 (33.8)	64 (12.5)	<0.001	3.56 (2.06-6.17)
**Outcomes**				
ICU stay (%)	63 (20.5)	116 (6.7)	<0.001	3.57 (2.55-4.99)
Need for ventilation	180 (58.8)	368 (21.7)	<0.001	5.16 (4.00-6.66)
**Time between events**				

	Dead<br>309 (15.1%)	Alive<br>1740 (84.9%)	p value	Crude OR (IC95%)
Days from admission to ICU, mean ± SD	4.5 ± 4.4	3.4 ± 2.4	0.050	1.10 (1.00-1.22)
Days from admission to ICU >7	8 (12.7)	6 (5.2)	0.073	2.67 (0.882-8.07)
Days from onset to admission, mean ± SD	5.0 ± 3.5	6.8 ± 13.3	0.142	0.98 (0.96-1.00)
Days from onset to ICU, mean ± SD	9.9 ± 5.7	9.8 ± 3.8	0.873	1.00 (0.94-1.08)
Days of ICU stay, mean ± SD	26.2 ± 18.0	22.8 ± 21.2	0.304	1.00 (0.99-1.02)
Days of stay, mean ± SD	14.8 ± 14.1	11.8 ± 14.6	0.006	1.01 (1.00-1.20)
Days of stay > 7	193 (65.2)	1055 (60.7)	0.142	1.21 (0.94-1.57)
**Underlying conditions**				
Number of chronic diseases, mean ± SD	2.9 ± 5.3	2.5 ± 1.8	<0.001	1.50 (1.37-1.64)
Anaemia	50 (17.0)	191 (10.9)	<0.008	1.58 (1.12-2.21)
Asthma	26 (8.8)	145 (8.3)	0.941	1.02 (0.66-1.57)
Body mass index (BMI), mean ± SD	29.3 ± 5.3	29.4 ± 5.8	0.855	1.00 (1.00-1.00)
Cancer	49 (16.7)	112 (6.4)	<0.001	2.76 (1.92-3.95)
Dementia	68 (22.2)	129 (7.4)	<0.001	3.66 (2.65-5.06)
Diabetes	99 (33.6)	386 (22.1)	<0.001	1.75 (1.34-2.27)
Heart disease	115 (39.1)	289 (16.5)	<0.001	3.15 (2.43-4.10)
Hypertension	195 (66.1)	780 (44.6)	<0.001	2.40 (1.87-3.10)
Ictus	36 (12.2)	88 (5.0)	<0.001	2.49 (1.66-3.75)
Immunodeficiency	10 (3.4)	29 (1.7)	0.066	1.98 (0.96-4.11)
Kidney disease	59 (20.1)	152 (8.7)	<0.001	2.63 (1.88-3.65)
Lung disease	59 (20.0)	157 (9.0)	<0.001	2.74 (1.99-3.78)
Liver disease	14 (4.6)	73 (4.2)	0.773	1.09 (0.61-1.96)
Neuromuscular disorders	27 (9.2)	83 (4.7)	<0.005	1.92 (1.22-3.02)
Obesity (BMI ≥ 30)	82 (46.6)	465 (48.6)	0.617	0.92 (0.67-1.27)
Rheumatic disease	109 (36.9)	392 (22.4)	<0.001	2.55 (1.84-3.54)
**Symptoms at admission**				
Abdominal pain	7 (2.3)	52 (3.0)	0.484	0.75 (0.39-1.67)
Ageusia	11 (3.6)	147 (8.6)	<0.004	0.40 (0.21-0.75)
Anosmia	10 (3.3)	159 (9.3)	<0.001	0.33 (0.17-0.64)
Chest pain	15 (4.9)	248 (14.4)	<0.001	0.31 (0.18-0.52)
Chills	10 (3.3)	57 (3.3)	0.963	0.98 (0.50-1.95)
Confusion	26 (8.5)	50 (2.9)	<0.001	3.11 (1.90-5.08)
Coryza	7 (2.3)	60 (3.5)	0.280	0.65 (0.29-1.43)
Cough	157 (51.5)	1277 (74.0)	<0.001	0.37 (0.29-0.48)
Diarrhoea	33 (10.7)	334 (19.4)	<0.001	0.50 (0.34-0.73)
Dizzy	13 (14.1)	79 (4.6)	0.791	0.92 (0.51-1.68)
Dyspnoea	203 (66.3)	1130 (65.4)	0.758	1.04 (0.81-1.35)
Fever	78 (25.4)	602 (34.9)	<0.001	0.63 (0.48-0.84)
Feverish	185 (60.1)	1179 (68.3)	0.005	0.70 (0.54-0.89)
General deterioration	116 (37.9)	554 (32.2)	0.049	1.29 (1.00-1.66)

	**Dead** 309 (15.1%)	**Alive** 1740 (84.9%)	**p value**	**Crude OR (IC95%)**
Headache	20 (6.5)	307 (17.8)	<0.001	0.32 (0.20-0.52)
Malaise	189 (61.8)	1059 (61.4)	0.902	1.02 (0.79-1.30)
Myalgia	40 (13.1)	441 (25.6)	<0.001	0.44 (0.31-0.62)
Nausea	18 (5.9)	141 (8.2)	0.169	0.70 (0.42-1.16)
Sore throat	6 (2.0)	96 (5.6)	0.011	0.34 (0.15-0.78)
Tachycardia	152 (49.4)	420 (24.3)	<0.001	3.03 (2.36-3.89)
Vomiting	9 (2.9)	121 (7.0)	0.009	0.40 (0.20-0.80)
**Clinical measurements**				
SBP (mm Hg), mean ± SD	127.7 ± 24.5	129.2 ± 20.5	0.273	0.99 (0.99-1.00)
SBP < 90 mm Hg	16 (5.5)	26 (1.7)	<0.001	3.44 (1.82-6.50)
DBP (mm Hg), mean ± SD	70.9 ± 14.8	75.1 ± 13.0	<0.001	0.97 (0.96-0.98)
DBP < 60 mm Hg	67 (22.9)	161 (10.2)	<0.001	2.60 (1.89-3.57)
Heart rate (bpm), mean ± SD	89.8 ± 19.8	91.2 ± 21.4	0.301	0.99 (0.99-1.00)
Long QT on the ECG	10 (8.2)	40 (6.2)	0.402	1.36 (0.66-2.80)
Oxygen saturation (%),mean ± SD	87.4 ± 8.1	93.0 ± 4.8	<0.001	0.87 (0.85-0.889)
Low oxygen saturation	223 (87.1)	910 (58.1)	<0.001	4.88 (0.34-7.13)
Respiratory rate (rpm), mean ± SD	25.3 ± 6.1	25.9 ± 29.5	0.870	0.99 (0.99-1.01)
**Biochemical alterations**				
Hypoalbuminaemia (albumin < 3.5 g/dl)	125 (52.7)	344 (24.1)	<0.001	3.52 (2.65-4.67)
ALT > 35 UI/l	74 (24.5)	601 (35.3)	<0.001	0.60 (0.45-0.79)
AST > 35UI/l	152 (52.2)	765 (46.0)	0.050	1.28 (1.00-1.65)
Hyperbilirubinaemia >1.2 mg/dl	25 (8.3)	62 (3.7)	<0.001	2.36 (1.46-3.82)
C-reactive protein > 1 mg/dl	301 (99.7)	1682 (98.4)	0.123	4.83 (0.65-35.70)
CPK > 145 IU/L	77 (37.9)	289 (23.5)	<0.001	1.99 (1.46-2.73)
D-dimer (> 500 µg/ml	233 (81.5)	1093 (65.6)	<0.001	2.30 (1.68-3.15)
Eosinophilia (> 500 eosinophils/µl)	9 (3.0)	83 (4.9)	0.160	0.61 (0.30-1.22)
Ferritin > 200 ng/ml	296 (100.0)	1673 (99.5)	0.992	1.11^{e+7} (0-Inf)
GGT > 38 UI/l	149 (50.3)	977 (58.3)	0.011	0.73 (0.57-0.93)
LDH > 247 UI/L	235 (84.2)	1226 (76.3)	0.004	1.66 (1.18-2.33)
Neutrophilopenia (< 1500 neutrophils/µl)	8 (2.6)	32 (1.9)	0.384	1.42 (0.65-3.10)
Neutrophilia (> 7700 neutrophils /µl)	109 (35.6)	264 (15.3)	<0.001	3.05 (2.34-3.99)
Thrombocytopenia (< 140 platelets /µl)	85 (28.0)	265 (15.4)	<0.001	2.13 (1.61-2.83)
Thrombocytophilia (> 370 platelets /µl)	303 (99.7)	1716 (99.8)	0.845	1.05 (0.62-1.77)
Prothrombin time > 14.3 seconds	97 (33.2)	238 (14.3)	<0.001	2.99 (2.26-3.96)
Urea in blood > 20 mg/ml	303 (99.7)	1598 (94.0)	0.003	19.3 (2.69-139)

Data is expressed by absolute (n) and relative (%) frequencies for categorical variables, and by mean ($\bar{x}$) ± standard deviation (SD) for quantitative variables. Figures in bold point for significant differences (p value ≤ 0.05). Abbreviations: HUVN: Virgen de las Nieves University Hospital, ICU: Intensive Care Unit, SBP: systolic blood pressure, DBP: diastolic blood pressure, ECG: electrocardiogram, ALT: alanine transaminase, AST: aspartate transaminase, CPK: creatinine phosphokinase, GGT: gamma-glutamyl transferase, LDH: lactate dehydrogenase. The reference categoryfor age is 0-40 years.

Table S2. Factors associated with **ICU admission** in Spanish COVID-19 hospitalised patients (I-MOVE-COVID19 study)

	**Admitted to ICU** 179 (8.7%)	**Not admitted to ICU** 1856 (91.3%)	**p value**	**Crude OR (IC95%)**
**Sociodemographic and clinical factors**				
Age, grouped (0-39; 40-64; 65-84; 85+ years)			<0.001	0.08 (0.04-0.13)
Age 0-39 years	15 (8.4)	197 (10.6)		(reference)
Age 40-64 years	88 (49.2)	720 (38.8)	0.104	1.61 (0.91-2.84)
Age 65-85 years	76 (42.5)	641 (34.6)	0.132	1.56 (0.88-2.77)
Age 85+ years	0 (0.0)	297 (16.0)	0.966	1.14e-7 (0.00-inf)
Sex (men)	974 (52.5)	109 (60.9)	0.032	1.41 (1.03-1.93)
Centre (HUVN)	117 (65.4)	710 (38.3)	<0.001	3.05 (2.21-4.20)
COVID-19 vaccinated	11 (10.7)	203 (22.1)	0.009	0.42 (0.22-0.80)
Flu vaccinated	70 (8.9)	716 (39.0)	0.832	1.03 (0.75-1.42)
Number of previous hospitalisations (last year), mean ± SD	4.43 ± 2.51	2.36 ± 3.19	0.143	1.13 (0.96-1.33)
Number of previous medical visits (last year), mean ± SD	7.38 ± 6.55	6.66 ± 7.20	0.405	1.01 (0.98-1.04)
Previous medical visits last year >12	17 (21.5)	70 (13.9)	0.078	1.7 (0.94-3.07)
**Outcomes**				
Death	63 (35.2%)	245 (13.2)	<0.001	3.57 (2.55-4.99)
Need for ventilation	176 (98.9%)	369 (20.4)	<0.001	344 (84-1393)
**Time between events**				
Days from onset to admission, mean ± SD	7.1 ± 3.4	6.5 ± 13.0	0.578	1.00 (0.99-1.01)
Days of stay, mean ± SD	34.4 ± 24.4	10.1 ± 11.2	<0.001	1.12 (1.10-1.13)
Days of stay > 7	171 (98.3)	1066 (57.7)	<0.001	41.71 (13.3-131)
**Underlying conditions**				
Number of chronic diseases, mean ± SD	2.6 ± 1.5	2.7 ± 1.9	0.535	0.96 (0.86-1.08)
Anaemia	16 (9.0)	223 (12.0)	0.240	0.73 (0.43-1.24)
Asthma	14 (7.9)	154 (8.3)	0.855	0.95 (0.54-1.68)
Body mass index (BMI), mean ± SD	31.6 ± 5.9	29.2 ± 5.6	<0.001	1.00 (1.00-1.00)
Cancer	9 (5.1)	151 (8.1)	0.153	0.60 (0.30-1.21)
Dementia	0 (0.0)	195 (10.5)	0.973	7.35^{e-8} (0.00-Inf)
Diabetes	58 (32.6)	424 (22.9)	0.004	1.63 (1.17-2.27)
Heart disease	31 (17.6)	367 (19.8)	0.486	0.87 (0.58-1.30)
Hypertension	92 (51.7)	878 (47.4)	0.273	1.19 (0.87-1.62)
Kidney disease	14 (8.0)	196 (10.6)	0.277	0.73 (0.41-1.29)
Ictus	9 (5.1)	115 (6.2)	0.553	0.81 (0.40-1.63)
Immunodeficiency	3 (1.7)	36 (1.9)	0.819	0.87 (0.27-2.86)
Liver disease	8 (4.5)	78 (4.2)	0.844	1.08 (0.51-2.27)
Lung disease	23 (12.8)	192 (10.4)	0.301	1.28 (0.80-2.03)

	Admitted to ICU 179 (8.7%)	Not admitted to ICU 1856 (91.3%)	p value	Crude OR (IC95%)
Neuromuscular disorders	6 (3.4)	102 (5.5)	0.236	0.60 (0.26-1.39)
Obesity (BMI ≥ 30)	59 (62.1)	485 (47.0)	0.005	1.84 (1.20-2.84)
Rheumatic disease	34 (19.1)	462 (24.9)	0.086	0.71 (0.48-1.05)
**Symptoms at admission**				
Abdominal pain	10 (5.6)	48 (2.6)	0.027	2.20 (1.10-4.44)
Ageusia	10 (5.6)	149 (93.7)	0.239	0.67 (0.35-1.30)
Anosmia	9 (5.1)	160 (8.7)	0.096	0.56 (0.28-1.11)
Chest pain	21 (8.1)	239 (13)	0.649	0.90 (0.56-1.44)
Chills	6 (3.4)	61 (3.3)	0.965	1.02 (0.43-2.39)
Confusion	1 (0.6)	75 (4.1)	0.046	0.13 (0.02-0.96)
Coryza	2 (3.0)	65 (3.5)	0.105	0.31 (0.08-1.28)
Cough	127 (71.3)	1295 (70.4)	0.795	1.05 (0.75-1.47)
Diarrhoea	31 (17.4)	335 (18.2)	0.794	0.95 (0.63-1.42)
Dizzy	8 (4.5)	83 (4.5)	0.987	0.99 (0.47-2.09)
Dyspnoea	131 (73.2)	1192 (64.7)	0.024	1.49 (1.05-2.10)
Fever	61 (34.5)	615 (33.4)	0.780	1.05 (0.76-1.45)
Feverish	112 (62.6)	1244 (67.6)	0.174	0.80 (0.58-1.10)
Malaise	92 (52.0)	1149 (62.4)	0.007	0.65 (0.48-0.89)
General deterioration	55 (30.9)	613 (33.4)	0.507	0.89 (0.64-1.25)
Headache	30 (16.9)	295 (16.1)	0.758	1.07 (0.71-1.61)
Myalgia	52 (29.1)	428 (23.3)	0.086	1.35 (0.96-1.89)
Nausea	18 (10.1)	139 (7.6)	0.238	1.36 (0.81-2.29)
Sore throat	9 (5.1)	92 (5.0)	0.979	1.01 (0.50-2.04)
Tachycardia	50 (28.1)	517 (28.1)	0.991	1.00 (0.71-1.41)
Vomiting	12 (6.7)	118 (6.4)	0.868	1.05 (0.57-1.95)
**Clinical measurements**				
SBP (mm Hg), mean ± SD	126 ± 22.1	129 ± 21.0	0.067	0.99 (0.98-1.00)
SBP < 90 mm Hg	7 (4.5)	35 (2.1)	0.051	2.23 (0.98-5.12)
DBP (mm Hg), mean ± SD	73.3 ± 13.4	74.6 ± 13.4	0.270	0.99 (0.98-1.01)
DBP < 60 mm Hg	23 (14.7)	201 (11.8)	0.287	1.29 (0.81-2.05)
Heart rate (bpm), mean ± SD	93.0 ± 33.6	90.8 ± 19.7	0.228	1.00 (1.00-1.01)
Long QT on the ECG	7 (8.9)	43 (6.3)	0.380	1.45 (0.63-3.35)
Oxygen saturation (%),mean ± SD	87.6 ± 7.4	92.6 ± 5.4	<0.001	0.90 (0.88-0.92)
Low oxygen saturation	118 (89.4)	1008 (60.1)	<0.001	5.60 (3.19-9.83)
Respiratory rate (rpm), mean ± SD	26.4 ± 11.1	25.7 ± 29.3	0.852	1.00 (0.99-1.01)
**Biochemical alterations**				
Hypoalbuminaemia (albumin < 3.5 g/dl)	41 (25.5)	425 (28.4)	0.425	0.86 (0.59-1.25)
ALT > 35 UI/l	73 (41.2)	601 (33.1)	0.030	1.42 (1.03-1.94)
AST > 35UI/l	104 (59.8)	807 (45.7)	<0.001	1.77 (1.29-2.42)

	Admitted to ICU 179 (8.7%)	Not admitted to ICU 1856 (91.3%)	p value	Crude OR (IC95%)
Hyperbilirubinaemia >1.2 mg/dl	14 (8.0)	73 (4.1)	0.019	2.04 (1.12-3.69)
C-reactive protein > 1 mg/dl	175 (100)	1794 (98.5)	0.985	4.91^{e+6} (0.00-Inf)
CPK > 145 IU/L	54 (37.8)	310 (24.2)	<0.001	1.90 (1.32-2.73)
D-dimer (> 500 µg/ml	118 (70.2)	1200 (67.8)	0.523	1.12 (0.79-1.58)
Eosinophilia (> 500 eosinophils/µl)	162 (92.6)	1519 (83.5)	0.068	0.34 (0.11-1.08)
Ferritin > 200 ng/ml	174 (100.0)	1781 (99.6)	0.991	3.84^{e+6} (0.00-Inf)
GGT > 38 UI/l	121 (69.5)	999 (55.9)	<0.001	1.80 (1.29-2.52)
LDH > 247 UI/L	162 (94.7)	1291 (75.9)	<0.001	5.73 (2.90-11.31)
Neutrophilopenia (< 1500 neutrophils/µl)	2 (1.1)	38 (2.1)	0.395	0.54 (0.13-2.25)
Neutrophilia (> 7700 neutrophils /µl)	53 (29.8)	318 (17.3)	<0.001	2.02 (1.43-2.85)
Thrombocytopenia (< 140 platelets /µl)	30 (16.9)	319 (17.4)	0.879	0.97 (0.64-1.46)
Thrombocytophilia (> 370 platelets /µl)	176 (99.4)	1829 (99.8)	0.505	1.23 (0.66-2.29)
Prothrombin time > 14.3 seconds	25 (14.8)	306 (17.2)	0.430	0.84 (0.54-1.30)
Urea in blood > 20 mg/ml	175 (98.3)	1715 (94.5)	0.040	3.37 (1.06-10.7)

Data is expressed by absolute and relative (%) frequencies for categorical variables, and by mean ($\overline{X}$) ± standard deviation (SD) for quantitative variables. Figures in bold point for significant differences (p value ≤ 0.05). Abbreviations: HUVN: Virgen de las Nieves University Hospital, ICU: Intensive Care Unit, SBP: systolic blood pressure, DBP: diastolic blood pressure, ECG: electrocardiogram, ALT: alanine transaminase, AST: aspartate transaminase, CPK: creatinine phosphokinase, GGT: gamma-glutamyl transferase, LDH: lactate dehydrogenase. The reference category for age is 0-40 years.

Table S3. Factors associated with **ventilation** in Spanish COVID-19 hospitalised patients (I-MOVE-COVID19 study)

	With ventilation 548 (26.7%)	Without ventilation 1456 (73.3%)	p value	Crude OR (IC95%)
**Sociodemographic and clinical factors**				
Age, grouped (0-39; 40-64; 65-84; 85+ years)			**<0.001**	**0.25 (0.18-0.35)**
Age 0-39 years				(reference)
Age 40-64 years	506 (92.5)	1291 (88.7)	0.159	1.31 (0.99-1.92)
Age 65-85 years	**310 (56.7)**	**690 (47.4)**	**<0.001**	**1.98 (1.36-2.89)**
Age 85+ years	76 (13.9)	215 (14.8)	0.109	1.42 (0.93-2.19)
Sex (men)	**325 (59.3)**	**739 (50.3)**	**<0.001**	**1.41 (1.16-1.73)**
Centre (HUVN)	**362 (66.1)**	**427 (29.3)**	**<0.001**	**4.69 (3.80-5.78)**
COVID-19 vaccinated	**40 (14.8)**	**175 (23.2)**	**0.004**	**0.57 (0.39-0.83)**
Flu vaccinated	**258 (47.6)**	**511 (35.5)**	**<0.001**	**1.65 (1.35-2.02)**
Number of previous hospitalisations (last year), mean ± SD	3.1 ± 2.6	2.3 ± 3.4	0.282	1.08 (0.94-1.25)
Number of previous medical visits (last year), mean ± SD	**8.1 ± 8.0**	**6.2 ± 6.6**	**0.003**	**1.04 (1.01-1.06)**

	With ventilation 548 (26.7%)	Without ventilation 1456 (73.3%)	p value	Crude OR (IC95%)
Previous medical visits last year > 12	45 (22.5)	44 (11.4)	<0.001	2.25 (1.42-3.55)
**Outcomes**				
Deaths (%)	180 (32.8%)	126 (8.7%)	<0.001	5.16 (4.00-6.66)
ICU stay (%)	176 (32.3%)	2 (0.1%)	<0.001	344 (85-1393)
**Time between events**				
Days from onset to admission, mean ± SD	6.6 ± 22.6	6.6 ± 4.9	0.949	1.00 (0.99-1.00)
Days from onset to admission, mean ± SD	6.6 ± 22.6	6.6 ± 4.9	0.949	1.00 (0.99-1.00)
Days from admission to ICU, mean ± SD	3.7 ± 2.9	1.5 ± 0.7	0.292	2.45 (0.46-13.00)
Days from admission to ICU > 7	13 (7.4)	0 (0.0)	0.690	0.413 (0.02-9.04)
Days from onset to ICU, mean ± SD	9.6 ± 4.1	4.5 ± 3.5	0.089	1.63 (0.93-2.85)
Days of ICU stay, mean ± SD	23.6 ± 19.8	23.5 ± 3.5	0.996	1.00 (0.93-1.07)
Days of stay, mean ± SD	21.6 ± 23.6	8.8 ± 6.2	<0.001	1.15 (1.13-1.17)
Days of stay > 7	472 (87.7)	747 (51.5)	<0.001	6.74 (5.11-8.89)
Number of chronic diseases, mean ± SD	2.8 ± 1.7	2.7 ± 1.9	0.436	1.03 (0.95-1.11)
Number of chronic diseases, mean ± SD	2.8 ± 1.7	2.7 ± 1.9	0.436	1.03 (0.95-1.11)
**Underlying conditions**				
Anaemia	40 (7.3)	198 (13.6)	<0.001	0.50 (0.35-0.72)
Asthma	44 (8.1)	121 (8.3)	0.861	0.97 (0.68-1.39)
Body mass index, mean ± SD	30.9 ± 5.4	29.1 ± 5.8	<0.001	1.00 (1.00-1.00)
Cancer	57 (10.5)	99 (6.8)	0.007	1.60 (1.14-2.25)
Dementia	41 (7.5%)	144 (9.9%)	0.106	0.74 (0.52-1.07)
Diabetes	164 (30.0)	312 (21.4)	<0.001	1.57 (1.26-1.96)
Heart disease	125 (22.9)	266 (18.3)	0.020	1.33 (1.05-1.69)
Hypertension	286 (52.5)	667 (45.8)	0.008	1.30 (1.07-1.59)
Ictus	39 (7.2)	81 (5.6)	0.184	1.31 (0.88-1.94)
Immunodeficiency	7 (1.3)	32 (2.2)	0.193	0.58 (0.25-1.32)
Kidney disease	50 (9.2)	158 (10.9)	0.276	0.83 (0.59-1.16)
Liver disease	20 (3.7)	67 (4.6)	0.362	0.79 (0.47-1.31)
Lung disease	78 (14.3)	133 (9.1)	<0.001	1.65 (1.23-2.23)
Neuromuscular disorders	24 (4.4)	85 (5.8)	0.209	0.74 (0.47-1.18)
Obesity (BMI ≥ 30)	158 (64.8)	384 (43.9)	<0.001	2.34 (1.75-3.15)
Rheumatic disease	106 (19.4)	394 (27.1)	<0.001	0.65 (0.51-0.83)
**Symptoms at admission**				
Abdominal pain	15 (2.8)	42 (2.9)	0.870	0.95 (0.52-1.73)
Ageusia	29 (5.4)	127 (8.8)	0.012	0.59 (0.39-0.89)
Anosmia	27 (5.0)	138 (9.6)	0.001	0.50 (0.32-0.76)
Chest pain	56 (10.4)	198 (13.7)	0.047	0.73 (0.53-1.00)
Chills	17 (3.2)	48 (3.3)	0.849	0.95 (0.54-1.66)
Confusion	17 (3.1)	59 (4.1)	0.332	0.76 (0.44-1.32)

	**With ventilation** 548 (26.7%)	**Without ventilation** 1456 (73.3%)	**p value**	**Crude OR (IC95%)**
Coryza	8 (1.5)	59 (4.1)	0.006	0.35 (0.17-0.74)
Cough	370 (68.5)	1032 (71.3)	0.223	0.87 (0.71-1.08)
Diarrhoea	78 (14.4)	288 (19.9)	0.005	0.68 (0.52-0.89)
Dizzy	22 (4.1)	68 (4.7)	0.544	0.86 (0.53-1.40)
Dyspnoea	401 (74.0)	901 (62.2)	<0.001	1.73 (1.39-2.15)
Fever	182 (33.6)	485 (33.6)	0.974	1.00 (0.81-1.24)
Feverish	345 (63.7)	995 (68.8)	0.031	0.80 (0.65-0.98)
Malaise	267 (49.6)	966 (66.7)	<0.001	0.49 (0.40-0.60)
General deterioration	177 (32.7)	480 (33.2)	0.825	0.98 (0.79-1.21)
Headache	75 (13.9)	246 (17.0)	0.094	0.79 (0.60-1.04)
Myalgia	139 (25.7)	330 (22.9)	0.188	1.17 (0.93-1.47)
Nausea	36 (6.7)	121 (8.4)	0.203	0.78 (0.53-1.15)
Sore throat	20 (3.7)	80 (5.5)	0.098	0.66 (0.40-1.08)
Tachycardia	168 (30.9)	394 (27.2)	0.109	1.19 (0.96-1.48)
Vomiting	26 (4.8)	101 (7.0)	0.076	0.67 (0.43-1.04)
**Clinical measurements**				
SBP (mm Hg), mean ± SD	127.2 ± 22.4	129.8 ± 20.5	0.019	0.99 (0.-99-1.00)
SBP < 90 mm Hg	17 (3.4)	23 (1.7)	0.028	2.01 (1.07-3.80)
DBP (mm Hg), mean ± SD	72.4 ± 12.8	75.2 ± 13.6	<0.001	0.98 (0.98-0.99)
DBP < 60 mm Hg	69 (13.9)	152 (11.5)	0.153	1.25 (0.92-1.70)
Heart rate (bpm), mean ± SD	91.4 ± 24.0	91.1 ± 20.2	0.803	1.00 (1.00-1.01)
Long QT on the ECG	15 (6.6)	33 (6.4)	0.942	1.02 (0.54-1.92)
Oxygen saturation (%),mean ± SD	88.8 ± 6.9	93.3 ± 4.8	<0.001	0.87 (0.85-0.89)
Low oxygen saturation	356 (85.0)	757 (55.4)	<0.001	4.55 (3.41-6.08)
Respiratory rate (rpm), mean ± SD	25.0 ± 8.0	27.1 ± 39.2	0.483	1.00 (0.99-1.01)
**Biochemical alterations**				
Hypoalbuminaemia (albumin < 3.5 g/dl)	136 (27.9)	318 (28.1)	0.935	0.99 (0.78-1.25)
ALT > 35 UI/l	197 (36.5)	473 (33.3)	0.183	1.15 (0.94-1.42)
AST > 35UI/l	287 (53.6)	621 (45.2)	< 0.001	1.40 (1.15-1.72)
Hyperbilirubinaemia >1.2 mg/dl	33 (6.2)	51 (3.7)	0.017	1.73 (1.10-2.71)
C-reactive protein > 1 mg/dl	539 (99.8)	1401 (98.2)	0.024	10.00 (1.36-73.8)
CPK > 145 IU/L	155 (34.9)	205 (21.6)	<0.001	1.95 (1.52-2.50)
D-dimer (> 500 μg/ml	377 (72.2)	923 (66.5)	0.018	1.31 (1.05-1.63)
Eosinophilia (> 500 eosinophils/μl)	17 (3.2)	70 (4.9)	0.095	0.63 (0.37-1.08)
Ferritin > 200 ng/ml	536 (100.00)	1390 (99.5)	0.990	1.17^{e+7} (0.0-Inf)
GGT > 38 UI/l	333 (63.2)	768 (54.8)	<0.001	1.42 (1.15-1.74)
LDH > 247 UI/L	461 (90.7)	981 (72.8)	<0.001	3.66 (2.65-5.06)
Neutrophilopenia (< 1500 neutrophils/μl)	9 (1.7)	31 (2.2)	0.374	1.12 (0.87-1.45)
Neutrophilia (> 7700 neutrophils /μl)	159 (29.3)	207 (14.4)	<0.001	2.46 (1.95-3.12)

	**With ventilation** 548 (26.7%)	**Without ventilation** 1456 (73.3%)	**p value**	**Crude OR (IC95%)**
Thrombocytopenia (< 140 platelets /µl)	101 (18.7)	244 (17.0)	0.374	1.12 (0.87-1.45)
Thrombocytophilia (> 370 platelets /µl)	539 (99.6)	1436 (99.9)	0.309	0.38 (0.05-2.67)
Prothrombin time > 14.3 seconds	95 (18.3)	229 (16.4)	0.330	1.14 (0.88-1.48)
Urea in blood > 20 mg/ml	527 (97.8)	1334 (93.6)	<0.001	3.00 (1.63-5.52)

Data is expressed by absolute and relative (%) frequencies for categorical variables, and by mean ($\overline{X}$) ± standard deviation (SD) for quantitative variables. Figures in bold point for significant differences (p value ≤ 0.05). Abbreviations: HUVN: Virgen de las Nieves University Hospital, ICU: Intensive Care Unit, SBP: systolic blood pressure, DBP: diastolic blood pressure, ECG: electrocardiogram, ALT: alanine transaminase, AST: aspartate transaminase, CPK: creatinine phosphokinase, GGT: gamma-glutamyl transferase, LDH: lactate dehydrogenase. The reference age category is 0-40 years
